# Relationships Between Muscle Parameters and History of Falls and Fractures in the Hertfordshire Cohort Study: Do All Muscle Components Relate Equally to Clinical Outcomes?

**DOI:** 10.1007/s00223-022-00986-w

**Published:** 2022-05-19

**Authors:** Faidra Laskou, Leo D. Westbury, Nicholas R. Fuggle, Mark H. Edwards, Cyrus Cooper, Elaine M. Dennison

**Affiliations:** 1 MRC Lifecourse Epidemiology Centre, University of Southampton, Southampton, UK; 2 NIHR Southampton Biomedical Research Centre, University of Southampton, Southampton, UK; 3 The Alan Turing Institute, London, UK; 4 Queen Alexandra Hospital, Portsmouth, UK; 5 NIHR Oxford Biomedical Research Centre, University of Oxford, Oxford, UK; 6 Victoria University of Wellington, Wellington, New Zealand

**Keywords:** Sarcopenia, Falls, Fractures, Muscle mass, Muscle strength, Gait speed

## Abstract

In previous work, relationships between muscle and bone size and strength have been demonstrated and were stronger in females, suggesting possible sexual dimorphism. Here we examine sex-specific associations between individual muscle sarcopenia components with clinical outcomes (falls and fractures). 641 participants were recruited. Muscle mass was assessed as cross-sectional area (CSA) by peripheral quantitative computed tomography of the calf, grip strength (GpS) by Jamar dynamometry and function by gait speed (GtS). Falls and fractures were self-reported. Ordinal and logistic regression were used to examine the associations between muscle measurements and outcomes with and without adjustment for confounders. Mean (SD) age was 69.3 (2.6) years. CSA, GpS, and GtS were greater among males (*p* < 0.002). A higher proportion of females had fallen since age 45 (61.3% vs 40.2%, *p* < 0.001); in the last year (19.9% vs 14.1%, *p* = 0.053); and reported a previous fracture since age 45 (21.8% vs 18.5%, *p* = 0.302), than males. Among females, greater CSA was related to reduced risk of falling and fewer falls in the previous year in fully adjusted analysis only (*p* < 0.05); higher GpS was related to lower risk of falls since age 45 in unadjusted analysis (*p* = 0.045) and lower risk of fracture since age 45 in both unadjusted and fully adjusted analysis (*p* < 0.045). No statistically significant associations were observed for GtS among either sex for any relationships between muscle measurements and clinical outcomes studied. We observed relationships between muscle mass and strength but not function with falls and fractures in females only; further longitudinal studies are required to reproduce these results.

## Introduction

Falls constitute a major risk factor for fracture and associated morbidity, mortality and economic costs [1]. Sarcopenia is an important contributor to falls risk, and hence fractures [2]. We have previously demonstrated relationships between muscle size and grip strength, and bone size and strength, supporting a role for the muscle-bone unit [3], with stronger relationships in females as it has been observed elsewhere [4, 5]. In 2019, the revised European Working Group on Sarcopenia in Older People 2 guidelines were published emphasising muscle strength, relative to muscle mass and function [6]. The aim of this study was to examine the strength of sex-specific associations between each of the key individual sarcopenia components (muscle mass, strength, and function) with the clinically important outcomes of falls and fractures in a population-based cohort of older adults.

## Materials and Methods

### The Hertfordshire Cohort Study

The Hertfordshire Cohort Study (HCS) comprises 2997 individuals born in Hertfordshire from 1931 to 1939 who lived there in 1998–2004 where they completed a home interview and clinic visit for a detailed health assessment. In 2004, of the 966 participants from the geographic region of East Hertfordshire who formed the in-depth musculoskeletal subgroup, 642 attended a clinic visit as part of a musculoskeletal follow-up study. The HCS baseline investigations had ethical approval from the Hertfordshire and Bedfordshire Local Research Ethics Committee and all participants provided written informed consent [7]; ethical approval was also obtained for all HCS follow-up studies. Further details of HCS have been described previously [7].

### Ascertainment of Participant Information in 1998–2004

Physical activity (Dallosso questionnaire) was ascertained by a nurse-administered questionnaire [8]. Dietary calcium intake was determined using a food-frequency questionnaire [9]. Current or most recent full-time occupation (husband’s for ever-married females) was ascertained. Social class was coded from the 1990 OPCS Standard Occupational Classification (SOC90) unit group for occupation [10], using computer-assisted standard occupational coding to generate the following occupational classes: I (Professional); II (Managerial and technical); IIINM (Skilled non-manual); IIIM (Skilled manual); IV (Partly skilled); V (Unskilled) [11]. These were dichotomised as follows: ‘Non-manual’ (I, II and IIINM) and ‘Manual’ (IIIM, IV and V). Fractures since age 45 years were self-reported. Among females, information on hormone replacement therapy (HRT) use, the age at which they had their last menstrual cycle and whether they had undergone a hysterectomy was also collected.

### Ascertainment of Participant Information in 2004–2005

Information on fractures since baseline, whether participants had fallen since age 45 years, the number of falls in the last year, smoking status and alcohol consumption was ascertained by a nurse-administered questionnaire. History of fracture since age 45 was determined from questionnaire data here and at baseline. Among females, information on HRT use was updated. Height was measured (Harpenden pocket stadiometer, Chasmors Ltd, London, UK) along with weight (SECA floor scale, Chasmors Ltd, London, UK) and used to derive BMI (kg/m^2^). Grip strength was measured three times for each hand using a Jamar dynamometer; the highest measurement was used for analysis. Customary gait speed in metres per second was calculated using a 3 m walk test. Radial and tibial (non-dominant side) peripheral qualitative computed tomography (pQCT) scans (Stratec 2000XL instrument, version 6.00) were performed; the other side was scanned if the non-dominant side had sustained a fracture. Calf muscle area was derived using default procedures, thresholds, and edge tracking settings to segment muscle from subcutaneous fat. Additional details relating to the pQCT scans have been published previously [12]. At time of assessment of the muscle size, strength, and function measures in this study (2004–2005), 33 (5%) participants were taking bisphosphonates and 113 (18%) were taking medications for the endocrine system. Associations of interest were similar if binary variables for current use of bisphosphonates and medications for the endocrine system were included as additional adjustments as shown in Table 2.

### Statistical Methods

Participant characteristics were described using summary statistics. Associations between calf muscle area, grip strength and gait speed in relation to binary outcomes were examined using logistic regression with and without adjustment for age, BMI, social class, smoker status, alcohol consumption, physical activity, dietary calcium intake, hormone replacement therapy use (females only) and time since menopause (females only), use of bisphosphonates and use of medications for the endocrine system. Relationships between predictors and number of falls in the last year (0, 1, > 1) were examined using ordinal regression with the same set of adjustments. Sex-stratified analyses were performed; *p* < 0.05 was regarded as statistically significant. Analyses were conducted using Stata, release 17.0. The analysis sample comprised 641 participants with data on at least one predictor and at least one outcome; of the 642 participants who attended the 2004–2005 follow-up stage, one participant had missing values for grip strength, gait speed and calf muscle area so they were excluded from the analysis sample.

## Results

### Descriptive Statistics

Participant characteristics of the analysis sample are presented in Table 1. Mean (SD) age was 69.3 (2.6) years. Calf muscle area, grip strength and gait speed were greater among males than females (*p* < 0.002 for all associations). Compared to males, a greater proportion of females had fallen since age 45 years (61.3% vs 40.2%, *p* < 0.001); fallen in the last year (19.9% vs 14.1%, *p* = 0.053); and had a previous fracture since age 45 years (21.8% vs 18.5%, *p* = 0.302). However, these latter two sex-differences were not statistically significant.

**Table 1:** Participant characteristics of the analysis sample

Characteristic	Males (*n* = 322)			Females (*n* = 319)			*P*-value
	Total N	Mean	SD	Total N	Mean	SD	
Age (years)	322	69.2	2.5	319	69.5	2.6	0.127
Height (cm)	322	173.7	6.7	319	160.5	6.1	< 0.001
Weight (kg)	322	82.3	12.4	319	71.7	13.8	< 0.001
BMI (kg/m^2^)	322	27.3	3.8	319	27.8	4.9	0.106
Dallosso activity score^a^	322	63.9	14.3	319	61.8	14.3	0.060
Calf muscle area (mm^2^)	293	8035	1204	295	6212	981	< 0.001
Grip strength (kg)	321	42.2	7.6	318	24.9	5.8	< 0.001
Gait speed (m/s)	320	0.92	0.17	317	0.88	0.16	0.001
	Total N	Median	IQR	Total N	Median	IQR	
Dietary calcium (g/day)^a^	322	1.2	1.0, 1.4	319	1.1	0.9, 1.3	< 0.001
Alcohol intake (units/week)	322	7.6	1.5, 16.5	317	1.3	0.0, 4.8	< 0.001
	Total N	N	%	Total N	N	%	
Smoker status	322			316			< 0.001
Never		121	37.6		200	63.3	
Ex		174	54		99	31.3	
Current		27	8.4		17	5.4	
Social class (manual)^b^	306	175	57.2	319	182	57.1	0.973
HRT use				319			N/A
Never					185	58	
At least 5 years ago					74	23.2	
Within last 5 years					47	14.7	
Current					13	4.1	
Years since menopause				316			N/A
< 10 years					11	3.5	
≥ 10 and < 15 years					49	15.5	
≥ 15 and < 20 years					75	23.7	
≥ 20 and < 25 years					60	19	
≥ 25 and < 30 years					33	10.4	
≥ 30 years					9	2.8	
Hysterectomy					79	25	
Fallen since age 45 years	321	129	40.2	318	195	61.3	< 0.001
Fallen in last year	319	45	14.1	317	63	19.9	0.053
Number of falls in last year	318			317			0.112
0		274	86.2		254	80.1	
1		36	11.3		49	15.5	
2 or more		8	2.5		14	4.4	
Fracture since age 45 years	314	58	18.5	317	69	21.8	0.302

^a^Ascertained at HCS baseline (1998–2004); all other characteristics were ascertained in 2004–2005

^b^Manual occupations comprise IIIM (Skilled manual), IV (Partly skilled) and V (Unskilled) from the 1990 OPCS Standard Occupational Classification (SOC90) unit group for occupation

*P*-values for sex-differences in characteristics were calculated using *t* tests, Wilcoxon rank-sum tests, or chi-squared tests as appropriate

### Relationships Between Muscle Size, Strength, and Function In Relation tTo Falls and Fractures

Associations between predictors (calf muscle area, grip strength, gait speed) and outcomes (fallen since age 45, fallen in last year, number of falls in last year, fracture since age 45) are presented in Table 2. Among females, greater calf muscle area was related to reduced risk of falling in the previous year and fewer falls in the previous year (*p* < 0.05) but only in fully adjusted analysis; higher grip strength was related to lower risk of falls since age 45 in unadjusted analysis only (odds ratio per SD greater grip strength: 0.79 (0.63, 0.99), *p* = 0.045) and lower risk of fracture since age 45 in both unadjusted (0.74 (0.56, 0.97), *p* = 0.030) and fully adjusted analysis (0.74 (0.56, 0.99), *p* = 0.044). No statistically significant associations were observed for gait speed among females, or among males for any of the predictors in relation to any of the outcomes.

**Table 2:** Odds ratios for outcomes per SD increase in predictors among males and females

P-ValuePredictor	Outcome	Males							*P*-Value
		Unadjusted		Adjusted*		Unadjusted			
		OR (95% CI)	*P*-value	OR (95% CI)	*P*-value	OR (95% CI)	*P*-value	OR (95% CI)	
Calf muscle area	Fallen since 45	0.97 (0.77, 1.23)	0.798	1.06 (0.79, 1.44)	0.691	0.93 (0.73, 1.17)	0.534	0.79 (0.58, 1.06)	0.119
	Fallen in last year	1.01 (0.72, 1.42)	0.941	1.13 (0.74, 1.72)	0.586	0.79 (0.59, 1.06)	0.120	**0.66 (0.44, 0.97)**	**0.037**
	No. falls in last year	1.04 (0.74, 1.47)	0.823	1.11 (0.72, 1.69)	0.643	0.79 (0.59, 1.06)	0.112	**0.64 (0.43, 0.95)**	**0.025**
	Fracture since 45	0.95 (0.70, 1.28)	0.722	0.96 (0.65, 1.42)	0.840	1.03 (0.78, 1.36)	0.838	1.11 (0.78, 1.58)	0.552
Muscle (Grip) strength	Fallen since 45	0.85 (0.68, 1.07)	0.167	0.87 (0.67, 1.12)	0.273	**0.79 (0.63, 0.99)**	**0.045**	0.79 (0.61, 1.01)	0.060
	Fallen in last year	0.75 (0.55, 1.03)	0.078	0.76 (0.54, 1.08)	0.129	0.88 (0.67, 1.17)	0.382	0.82 (0.60, 1.11)	0.198
	No. falls in last year	0.77 (0.56, 1.06)	0.105	0.78 (0.55, 1.11)	0.175	0.85 (0.64, 1.13)	0.273	0.77 (0.57, 1.06)	0.109
	Fracture since 45	1.33 (0.98, 1.81)	0.070	1.35 (0.95, 1.92)	0.098	**0.74 (0.56, 0.97)**	**0.030**	**0.74 (0.55, 0.99)**	**0.042**
Gait speed	Fallen since 45	0.99 (0.79, 1.23)	0.902	1.00 (0.78, 1.28)	0.988	0.85 (0.68, 1.07)	0.173	0.87 (0.67, 1.13)	0.309
	Fallen in last year	0.76 (0.55, 1.05)	0.100	0.83 (0.58, 1.17)	0.287	0.88 (0.67, 1.16)	0.352	0.87 (0.63, 1.19)	0.374
	No. falls in last year	0.77 (0.55, 1.07)	0.124	0.83 (0.58, 1.18)	0.294	0.84 (0.64, 1.12)	0.232	0.84 (0.61, 1.16)	0.289
	Fracture since 45	1.16 (0.87, 1.54)	0.301	1.10 (0.80, 1.52)	0.547	1.06 (0.81, 1.39)	0.688	1.09 (0.80, 1.47)	0.593

*OR* Odds ratio; *CI* confidence interval; *SD* standard deviation

Sex-specific z-scores were derived for calf muscle area, grip strength and gait speed to enable the comparison of effect sizes

^*^Adjusted for age, BMI, social class, smoker status, alcohol consumption, physical activity (ascertained from 1998 to 2004), dietary calcium intake (ascertained from 1998–2004), hormone replacement therapy use (females only), time since menopause (females only), use of bisphosphonates and use of medications for the endocrine system

Odds ratios for being in a higher category for number of falls in the last year (0, 1 or > 1) were estimated using ordinal regression; logistic regression was used for the other outcomes

All participant characteristics were ascertained from 2004 to 2005 unless stated otherwise

## Discussion

In this study, higher grip strength was related to lower risk of falls and fractures since age 45 years and greater muscle size was associated with both reduced risk of falling and fewer falls in the previous year. The association between muscle strength and risk of fractures remained robust after adjustments. Conversely, associations regarding muscle size were only significant in adjusted models. Our findings support previous evidence that muscle strength is a key characteristic in detecting older adults at risk of adverse outcomes including falls and fractures [6]. Our study once again demonstrated sexual dimorphism in relationships observed and in general accord with previous literature, although previous studies have also suggested important relationships between muscle measures and bone outcomes in men [5].

Gait speed was not associated with prevalent falls and fractures in this study. Gait speed has been shown to reflect health and functional status, and to be associated with survival in older adults [13–15]. We previously found no associations of gait speed with measures of bone size, strength and density in the same cohort [3]. Amongst other physical performance tests, gait speed has previously been shown to be weakly associated with risk of hip fractures in participants without walking difficulties [16]. Gait speed is suitable for screening of poor physical performance and is used to identify cases of severe sarcopenia, as defined by the European Working Group on Sarcopenia in older adults (EWSGOP2) [6], but it is possible that it is more adversely affected by gait ability and/or severe weakness that leads to falls and fractures [17]. Two main types of gait speed assessment exist: the short-and long-distance gait test. Some groups favour the use of long-distance gait speed for its established relationship to mobility disability and public health relevance [18, 19]. Conversely, short gait tests can be used as surrogates for long-distance speed tests for the assessment of functional status in older adults, and are easily implemented into clinical practice [6, 20]. Thus, we suggest that gait speed combined with other physical performance measures, such as chair stand test, might perform better as a predictor of falls and fractures when assessing community-dwelling older adults [16].

There are several strengths and limitations to this work which was undertaken in a very well-characterised cohort that has previously been shown to be representative of the UK population [7]. While the sex differences noted in our study insights into potential differential sex-specific mechanisms, a healthy bias in males, as indicated by the relatively higher mean of grip strength and gait speed, and the use of specific cut-off points to define each sarcopenia components should also be considered as a contributing factor to the absence of associations between sarcopenia and falls and/or fractures in males. However, since the cohort is made up of community-dwelling individuals, generalisability of these findings to less healthy or institutionalized groups may be limited. Specifically, we also acknowledge the limitations associated with self-reported outcomes and the need for prospective data.

## Conclusion

In conclusion we have observed relationships between muscle mass and strength but not function with falls and fractures in females but not males. Large prospective studies are needed to confirm the above-mentioned relationships, and to further explore the sexual dimorphism observed.
